# Unilateral Lateral Entorhinal Inactivation Impairs Memory Expression in Trace Eyeblink Conditioning

**DOI:** 10.1371/journal.pone.0084543

**Published:** 2013-12-19

**Authors:** Stephanie E. Tanninen, Mark D. Morrissey, Kaori Takehara-Nishiuchi

**Affiliations:** 1 Department of Psychology, University of Toronto, Toronto, Ontario, Canada; 2 Program in Neuroscience, University of Toronto, Toronto, Ontario, Canada; 3 Department of Cell and Systems Biology, University of Toronto, Toronto, Ontario, Canada; Federal University of Rio Grande do Norte, Brazil

## Abstract

Memory in trace eyeblink conditioning is mediated by an inter-connected network that involves the hippocampus (HPC), several neocortical regions, and the cerebellum. This network reorganizes after learning as the center of the network shifts from the HPC to the medial prefrontal cortex (mPFC). Despite the network reorganization, the lateral entorhinal cortex (LEC) plays a stable role in expressing recently acquired HPC-dependent memory as well as remotely acquired mPFC-dependent memory. Entorhinal involvement in recent memory expression may be attributed to its previously proposed interactions with the HPC. In contrast, it remains unknown how the LEC participates in memory expression after the network disengages from the HPC. The present study tested the possibility that the LEC and mPFC functionally interact during remote memory expression by examining the impact of pharmacological inactivation of the LEC in one hemisphere and the mPFC in the contralateral hemisphere on memory expression in rats. Memory expression one day and one month after learning was significantly impaired after LEC-mPFC inactivation; however, the degree of impairment was comparable to that after unilateral LEC inactivation. Unilateral mPFC inactivation had no effect on recent or remote memory expression. These results suggest that the integrity of the LEC in both hemispheres is necessary for memory expression. Functional interactions between the LEC and mPFC should therefore be tested with an alternative design.

## Introduction

The entorhinal cortex (EC) has been implicated in the formation and expression of hippocampus-dependent memory as an interface between hippocampal and neocortical memory representations [1-3]. In trace eyeblink conditioning (TEBC), the EC is necessary for memory acquisition [4,5] as well as the expression of one-day-old (recent) and one-month-old (remote) memory [6]. The role of the EC in memory acquisition and initial expression is expected given its role as a primary input structure to the hippocampus, which is necessary for memory acquisition and initial retention [7-11]. In contrast, it remains unknown how the EC participates in the expression of remote memories that no longer require the hippocampus [7,9,10]. One possibility is that the lateral portions of the entorhinal cortex (LEC) interact with the prelimbic area of the medial prefrontal cortex (PrL), a region that plays a key role in the expression of remote memory [9,12-14].This idea is supported by their reciprocal monosynaptic connections [15-17] and synchronization of their theta oscillations during memory expression [18]; however, the necessity of the LEC-PrL connection for memory expression has not been tested. To test this, we reversibly disconnected the LEC from the PrL with an asymmetric inactivation technique [19] and examined its effect on the expression of recent and remote memory in TEBC. We found that inactivation of the LEC in one hemisphere combined with inactivation of the PrL in the contralateral hemisphere impaired memory expression at both recent and remote time points; however, unexpectedly, the degree of impairment was not significantly different from that with unilateral inactivation of the LEC alone. 

## Materials and Methods

### Subjects

Twenty-six male Long-Evans rats (Charles River Laboratories, St.-Constant, QC, Canada), 70 days old upon arrival, were individually housed in transparent plastic cages in a home colony room and maintained on a 12-hour reverse light/dark cycle (dark from 10:00 to 22:00) with free access to food and water. 

### Ethics Statement

All procedures were in accordance with the National Institutes of Health Guide for Care and Use of Laboratory Animals (Publication NO. 85-23, revised 1985), the Canadian Council on Animal’s Care, APA ethical standard, and approved by the University of Toronto Animal Care Committee. 

### Design

All animals experienced 10 daily acquisition sessions in trace eyeblink conditioning (TEBC). Memory performance was then tested with eight retention sessions either one day (Recent group) or one month (Remote group) after the last acquisition session. Once completed, some rats underwent three sessions of delay eyeblink conditioning (DEBC). 

### Surgery

**Figure 1:**
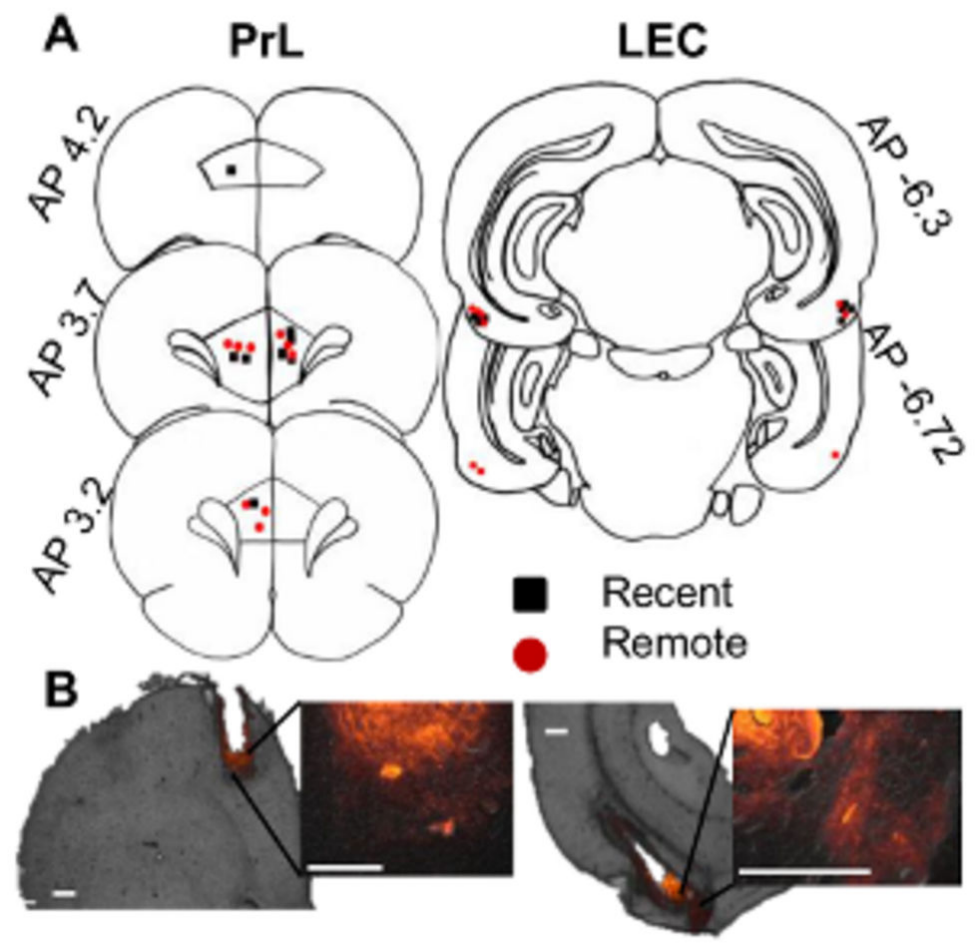
Cannulae locations and muscimol spread in the prefrontal cortex and lateral entorhinal cortex. **A**, The rats whose cannulae tips were located in the prelimbic area of the medial prefrontal cortex (PrL; left) and lateral entorhinal cortex (LEC; right) were included in the behavioral analyses (Recent group, black; Remote group, red). The hemisphere that was implanted with a cannula was counterbalanced across rats. **B**, The spread of muscimol was estimated with muscimol tagged to a fluorescent molecule (red). The spread was mostly restricted to the PrL (right) or LEC (left). The inset images show magnified views of each infusion site. Scale bars indicate 500 μm.

The rats experienced one of two surgery protocols: (1) the Recent group had guide cannulae and electromyogram (EMG) electrodes surgically implanted one week before acquisition sessions, and (2) the Remote group had EMG electrodes implanted before acquisition sessions and in an additional surgery one week before retention sessions, the EMG electrodes were replaced with new ones, and guide cannulae were also implanted. The replacement of EMG electrodes was necessary to obtain good signals during retention sessions because the quality of the EMG electrodes degrades during the month retention period. Under anesthesia (1-1.5% isoflurane by volume in oxygen at a flow rate of 1.5 L/min; Holocarbon Laboratories, River Edge, NJ, USA), four stainless steel wires attached to a connector cap were positioned subcutaneously in the upper left *orbicularis oculi* (eyelid muscle) to record EMG activity and deliver a periorbital shock. An additional reference wire was implanted in the neck or cheek muscle. The coordinates for cannula placements were chosen based on our previous studies that showed impairments in memory expression after bilateral inactivation of lateral portions of the entorhinal cortex (LEC; [6]) or the prelimbic region of medial prefrontal cortex (PrL; [14]). Small burr holes were drilled into the skull, and guide cannulae (Plastics One, Roanoke, VA, USA) were lowered into the LEC in one hemisphere [-6.45 anterioposterior (AP), ±6.65 mediolateral (ML), -8.5 dorsoventral (DV) millimeters from bregma] and the PrL in the contralateral hemisphere (3.2 AP, ±0.8 ML, -3.5 DV millimeters from bregma; Figure 1A). Left and right hemisphere cannulae positions were counterbalanced between rats. The EMG electrodes and guide cannulae were secured to the skull with stainless steel screws and dental acrylic resin, and guide cannulae were capped with dummy stylets. 

### Eyeblink Conditioning

#### Conditioning apparatus

**Figure 2:**
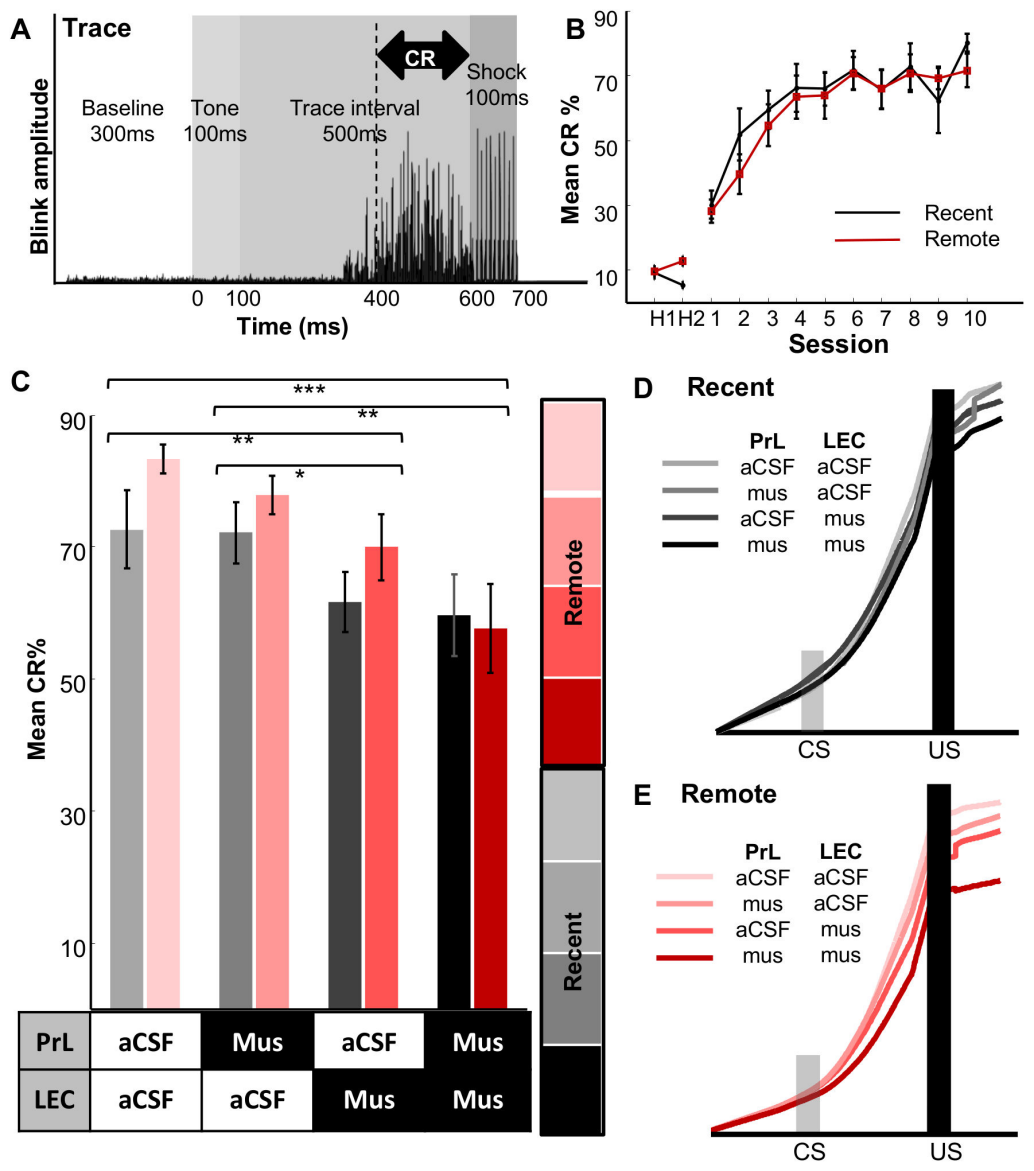
Unilateral inactivation of the lateral entorhinal cortex impairs recent and remote memory expression. **A**, During trace eyeblink conditioning sessions, the conditioned stimulus (CS; 100 ms tone) and unconditioned stimulus (US; 100 ms periorbital shock) were separated by a 500 ms stimulus-free trace interval. The conditioned response (CR) was detected as an increase in eyelid electromyogram activity prior to US onset (black marking, bottom). **B**, The rats received 10 days of acquisition followed by eight days of retention sessions either one day (Recent group, black, n = 8) or one month (Remote group, red, n = 9) after the last acquisition session. Following two habituation sessions (H1 and H2), both the Recent and Remote groups increased the frequency of CR expression (Mean CR %) in 10 sessions. **C**, The retention sessions were preceded by microinfusions of artificial cerebral spinal fluid (aCSF) or muscimol (Mus) into the prelimbic region of the prefrontal cortex (PrL) in one hemisphere or lateral entorhinal cortex (LEC) in the other hemisphere. Unilateral infusions of muscimol into the LEC significantly reduced CR% for the Recent (black) and Remote (red) groups. The CR% did not further decrease with additional muscimol infusions into the contralateral PrL. *p < .05; **p < .01; *** *p* < .001. **D**, **E**, The increase in CR amplitude following CS onset was smaller after muscimol infusions into the PrL and LEC (black or red line) than the other conditions in both Recent (D) and Remote (E) retention.

**Figure 3:**
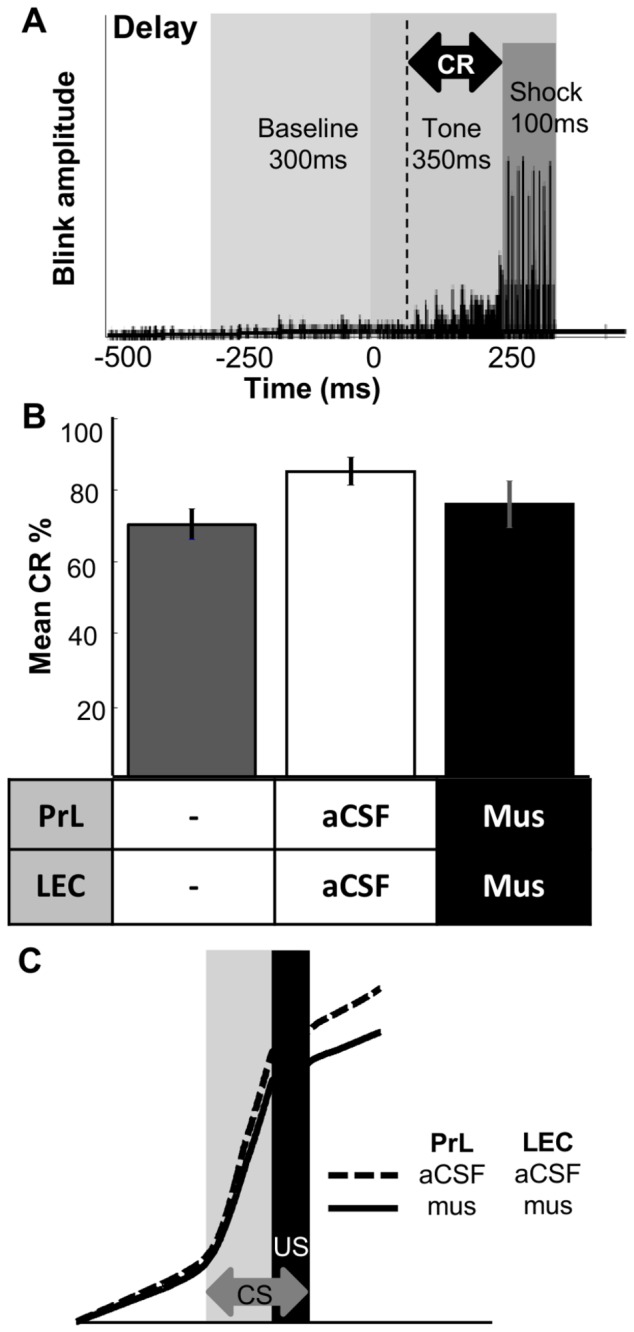
Prefrontal-entorhinal disconnection did not impair memory expression in delay eyeblink conditioning. **A**, During delay eyeblink conditioning sessions, the rats received the conditioned stimulus (CS; 350 ms tone) which co-terminated with the unconditioned stimulus (US; 100 ms periorbital shock). An increase in EMG activity before US onset was defined as a conditioned response (CR; black marking). **B**, After retention sessions, the Remote group was trained in delay eyeblink conditioning. The rats acquired the association in one session (grey bar), and the CR expression (CR %) was comparable after either artificial cerebral spinal fluid (aCSF; white bar) or muscimol (Mus; black bar) infusions into the prelimbic prefrontal cortex (PrL) and lateral entorhinal cortex (LEC). **C**, The increase in CR amplitude following CS onset was similar after aCSF or muscimol infusions.

Animals were placed into a transparent, cylindrical, plastic container and put into a sound and light-attenuating chamber. Stimulus delivery was controlled by a microcomputer (BasicX, Netmedia, Tucson, AZ, USA). The conditioned stimulus (CS) was a 100 ms tone (2.5 kHz, 85 dB) in TEBC (Figure 2A) or a 350 ms tone in DEBC (Figure 3A) delivered through a speaker in the chamber ceiling. The unconditioned stimulus (US) was a 100 ms periorbital shock (100 Hz square pulses) delivered to the eyelid with a stimulus isolator (ISO-Flex, A.M.P.I., Jerusalem, Israel). In TEBC, a 500 ms interval separated the CS and US, and in DEBC, the CS and US co-terminated. The shock level was initiated at 0.3 mA and was calibrated daily to induce an optimal unconditioned response (UR; an eyeblink/head-turn response) which was monitored through web-cameras mounted inside the chambers. The conditioned response (CR) was defined as eyeblink responses elicited during a 200-ms window immediately before US onset. Eyeblink responses were monitored by recording electromyogram (EMG) activity from the left upper *orbicularis oculi* through two surgically implanted stainless steel wires. EMG activity was band-pass filtered between 0.3 and 3.0 kHz, digitized at 6.0 kHz, and stored using a RZ-5 recording system (Tucker-Davis Technologies, Alachua, FL). 

#### EMG analysis

The analysis of EMG activity was the same as those used in our previous studies [6,9]. The amplitude of the EMG signal at time *t* was calculated by subtracting the minimum signal from the maximum signal during a 1 ms window. EMG amplitude was averaged during a 300 ms window before CS presentation in each trial and then averaged across all trials to set a threshold, its mean + one standard deviation. This threshold was subtracted from the EMG amplitudes in each trial and only amplitudes that exceeded this threshold were averaged during a 300 ms window before CS onset to produce a pre-value and during a 200 ms window before US onset to produce a CR value. This 200 ms CR value captures the adaptive blinking response that occurs immediately before US onset. A trial contained a CR when the CR value was five times larger than the pre-value. A trial was discarded when the rat's hyperactivity produced a pre-value that exceeded 30% of the threshold. The percent of conditioned responding (CR%) for each animal per session was the ratio of CR trials to the total number of valid trials. To determine whether the manipulations affected the temporal pattern of the CR, we visually inspected the integrated area of the CR which is calculated by cumulatively summing the averaged EMG amplitude minus the pre-value for all valid trials. 

#### Behavioral procedure

The rats were given at least three days to recover after EMG electrode surgeries and seven days after EMG electrode and cannulae implantation surgeries before beginning daily conditioning sessions. Each rat was placed in the chamber for 10 min to adapt to the surroundings and then underwent 50 min of TEBC. For sessions 1-2, the rats were placed in the conditioning chamber for 50 min in the absence of CS-US presentation to adapt to the procedures, during which eyelid EMG activity was recorded. CS-US presentation occurred during sessions 3-12 where the rats were presented with 90 CS-US pairings and 10 CS only trials (every tenth trial) over the course of 50 min. The inter-trial intervals were pseudo-randomized between 20 s and 40 s with a mean of 30 s. The rats that achieved a CR% of over 45% on the last three days of the acquisition sessions were then tested for their retention. We used 45% as our criterion for learning based on the distribution of the averaged CR% for the last 3 days of acquisition of all rats (data not shown); 45% was approximately 1.5 standard deviations from the mean CR%. During eight retention sessions they received microinfusions of either muscimol or artificial cerebral spinal fluid (aCSF) before the conditioning started. Subsequently, the rats received the same paired presentations of the CS and US as the acquisition sessions. The paired CS-US presentation has been a standard procedure to measure retention in TEBC [7,20-22]. It, however, may induce relearning, which may be confounded with the effect of drug. In fact, over the course of eight retention sessions, the CR% became significantly higher in the Recent and Remote groups with no differences between groups (two-way repeated measures ANOVA; Session F_7,105_ = 4.287, *p*<0.001; Group, F_1,15_ = 1.323, *p* = 0.268). To minimize this confounding factor of relearning, the order of manipulation types (i.e., either muscimol or aCSF into the PrL or LEC) was randomized across rats. 

Following the retention sessions, the Remote group experienced three days of DEBC where they were trained in the paradigm on the first day and received microinfusions of aCSF and muscimol on the second and third day, respectively. 

### Intracerebral Drug Infusion

Before each retention session, the rats were held in plastic restrainers (Stoelting, Wood Dale, IL, USA) and received microinfusions of either a muscimol solution (Muscimol HBr, Sigma-Aldrich, Oakville, ON, Canada; 0.5 µg in 0.5 µL aCSF) or aCSF into the LEC or PrL. In the LEC, 0.5 μL was infused at a rate of 0.5 μL/min and in the PrL, 0.3 μL was infused at a rate of 0.3 μL/min. Infusion volumes for the LEC were chosen based on a previous study [6]. We reduced the volume for the PrL from that used in previous studies [14,23] because unilateral infusions of 0.5 μL of muscimol reduced CR% down to ~35% (unpublished observation). Because 0.5 μL of muscimol solution spreads ~1 mm in diameter [24], the data suggested that the muscimol solution spread into the contralateral hemisphere, which was only ~0.5 mm away from the injection site. 

Immediately before a retention session, the rats received one of four patterns of manipulation: aCSF in both the LEC and PrL; aCSF in the LEC and muscimol in the PrL; muscimol in the LEC and aCSF in the PrL; or muscimol in both the LEC and PrL. The experimenter was blind to which drug the animal received. Over the course of eight retention sessions, each rat received each manipulation type twice; once during the first 4 sessions (1^st^ block) and once during the latter 4 sessions (2^nd^ block). The order of the four manipulations was randomized across rats in each block. An example retention schedule follows: BLOCK 1|Day 1 aCSF-PrL/muscimol-LEC; Day 2 aCSF-PrL/aCSF-LEC; Day 3 muscimol-PrL/muscimol-LEC; Day 4 muscimol-PrL/aCSF-LEC; BLOCK 2|Day 5 muscimol-PrL/muscimol-LEC; Day 6 aCSF-Prl/muscimol-LEC; Day 7 aCSF-PrL/aCSF-LEC; Day 8 muscimol-PrL/aCSF-LEC. The data with the same manipulation were averaged across blocks. This would reduce the confounding factor of forgetting and relearning. The animals were placed in the conditioning chamber at least 10 min after the infusions. They were also given 10 min of habituation to the chamber which resulted in at least 20 min to allow muscimol to spread to its maximal range [25] before retention sessions began. 

### Histology

After behavioral testing, each rat was given a lethal intraperitoneal injection of sodium pentobarbital (102 mg/kg; Euthansol, Merck, Kirkland, QC, Canada) and then perfused intracardially with 0.9% saline and 10% formalin. The brain tissue was then cryoprotected, sectioned coronally at 50 µm, mounted onto slides, and stained with cresyl violet. Cannula positions were located using a light microscope and mapped with the stereotaxic atlas of the rat brain [26]. To determine the spread of muscimol, four rats were given microinjections, prior to the perfusion procedure, of a conjugate of muscimol and the BODIPY ® TMR-X fluorophore (Molecular Probes, Eugene, OR, USA) into the LEC and PrL, with the same microinfusion procedure used during retention sessions. The spread of muscimol was detected with a fluorescent upright microscope (Axio Imager, Z1, Zeiss, Jena, Germany) with a DsRed filter cube. Pictures of the tissue were taken with a Retiga EX1 camera (Q-Imaging, Surrey, BC, Canada) and viewed with imaging software (Volocity, Perkin Elmer, Waltham, MA, USA). 

### Statistical Analyses

Data are expressed as the CR% of each session, which is the group's mean ± the group's standard error of the mean. Repeated-measures ANOVAs and paired sample *t*-tests with manipulation pattern as the within-subjects factor were used to determine statistical significance. Within-subjects contrasts were used as a *post-hoc* test where applicable. 

## Results

### Histology

Most cannulae were in their target positions in either the rostrolateral portions of the entorhinal cortex, typically defined as the lateral entorhinal cortex (LEC; [15]) or the prelimbic region of the medial prefrontal cortex (PrL; Figure 1A). Cannulae were misplaced in six rats, and their data were removed from the behavioral analyses. 

The spread of muscimol was confined to the dorsal part of the PrL or LEC (Figure 1B). Spreading into the contralateral PrL was not observed. In some cases, there was a marginal spread into the perirhinal cortex. 

### Effects of LEC-PrL Disconnection on Memory Expression in Trace Eyeblink Conditioning

The rats were trained in trace eyeblink conditioning (TEBC) followed by eight days of retention sessions beginning either one day (Recent group) or one month (Remote group) after the last acquisition session. After removing six rats because of improper cannulae location and three rats because of equipment dysfunction during a retention session, there were eight rats in the Recent group and nine rats in the Remote group. 

Over the course of acquisition sessions, the Recent and Remote groups increased the frequency of their expression of the conditioned response (CR%) with no differences between groups (Figure 2B, two-way repeated measures ANOVA; Session, F_9_,_135_ = 20.452, *p* < .001; Group, F_1,15_ = 0.206, *p* = .656). 

Immediately before the retention sessions, the Recent and Remote groups received one of four pharmacological manipulation patterns: muscimol in both the LEC and PrL, artificial cerebral spinal fluid (aCSF) in both the LEC and PrL, or aCSF in the LEC/PrL and muscimol in the other region (Figure 2C). A two-way mixed ANOVA revealed a difference between manipulations (Manipulation, F_3,45_ = 9.470, *p* < .001) but no differences in CR% between the Recent and Remote groups (Group, F_1,15_ = 1.323, *p* = .268). Accordingly, the two groups were collapsed together for all subsequent analyses. We first confirmed with Chi-square analyses that the manipulation patterns were randomized across days in the first block (X^2^_(9,17)_ = 6.353, *p* = .704) and the second block (X^2^_(9,17)_ = 4.471, *p* = .878). Next, we examined the differences between manipulation patterns; within subjects comparisons revealed differences in CR% during the sessions with muscimol in the LEC or muscimol in the LEC and PrL in comparison to sessions with aCSF in both regions (Manipulation, F_1,15_ = 9.109, *p* < .01; F_1,15_ = 31.15, *p* < .001 respectively) and in comparison to sessions with muscimol in the PrL only (Manipulation, F_1,15_ = 5.008, *p* = .04 and F_1,15_ = 13.182, *p* = .002 respectively). In addition, the sessions with muscimol in the LEC and muscimol in the LEC and PrL were not significantly different from each other (Manipulation, F_1,15_ = 2.565, *p* = .13). Furthermore, the sessions with aCSF in both regions was not significantly different from sessions with muscimol in the PrL only (Manipulation, F_1,15_ = 0.672, *p* = .425). The manipulations did not alter the timing of blinking during Recent (Figure 2D) or Remote retention (Figure 2E). 

### Effects of LEC-PrL Disconnection on Memory Expression in Delay Eyeblink Conditioning

Lastly, we assessed whether LEC-PrL disconnection produced any perceptual or motor deficits by examining its effect on memory expression in delay eyeblink conditioning (DEBC; Figure 3A). DEBC uses the same stimuli and motor outputs as those in TEBC but does not depend on the LEC [6] or the PrL [23]. As expected, CR% with muscimol infusions in the LEC and PrL was comparable to that with aCSF infusions (paired sample *t*-test, t_6_ = 1.368, *p* = .220; Figure 3B). In addition, the integrated CR during sessions with muscimol infusions was similar to that with aCSF infusions (Figure 3C). 

## Discussion

The present study was designed to determine whether the monosynaptic connection between the prelimbic region of the medial prefrontal cortex (PrL) and lateral entorhinal cortex (LEC) is necessary for the expression of recent or remote memory learned through trace eyeblink conditioning (TEBC). However, we were unable to detect a functional interaction with the current asymmetric disconnection design because unilateral LEC inactivation produced a significant memory impairment thereby masking the hypothesized effect of LEC-PrL disconnection. Nevertheless, the present study provides further support for a role of the LEC in memory expression regardless of time passage after learning [6] while demonstrating that memory expression depends on both hemispheres of the LEC, but not the PrL. 

Asymmetric inactivation capitalizes on the concept that unilateral inactivation of a brain region does not usually produce a significant effect on behavior. Consistent with this, unilateral inactivation of the PrL did not have any effect on memory expression in TEBC (Figure 2C). In contrast, unilateral inactivation of the LEC significantly impaired memory expression though the degree of impairment was less severe than that after bilateral inactivation [6]. Behavioral impairment after unilateral dysfunction has a precedent; unilateral LEC lesions reduced discrimination between novel object-context associations to a level comparable to bilateral lesions [27]. Similarly, unilateral inactivation of the ventral hippocampus (HPC) or amygdala, but not PrL, significantly impaired memory acquisition in trace fear conditioning [28]. The deficit in mnemonic processing after unilateral amygdala inactivation has been further corroborated in other studies (e.g., 29,30). In addition, unilateral inactivation of the dorsal hippocampus has impaired spatial memory (e.g., 31,32) and conditioned passive avoidance (e.g., 33). Together, these findings suggest that some brain structures may have more extensive inter-hemispheric interactions than others [34], and that these inter-hemispheric interactions may play a key role in some behaviors. 

In the present study, we expected LEC-PrL disconnection during remote retention to reduce CR% to the level that was observed with bilateral LEC inactivation (~45%, [6]) or substantially reduce to CR% to the level observed with bilateral PrL inactivation (~5%, [14]). However, LEC-PrL disconnection produced a modest impairment that was comparable to unilateral LEC inactivation. This finding raises the possibility that LEC-PrL disconnection may be circumvented by auxiliary pathways that can indirectly connect the LEC and PrL and ameliorate the effects of LEC-PrL disconnection. For example, the LEC may act on the ventral HPC which sends monosynaptic projections to the PrL [35,36]. Consistent with this idea, the involvement of the ventral HPC is implicated in the acquisition of trace fear conditioning [28,37,38]. Moreover, some neurons in the ventral HPC respond to the conditioned or unconditioned stimulus in TEBC but to a lesser degree than neurons in the dorsal HPC [39]. Alternatively, the PrL may be capable of interacting directly with the perirhinal cortex, a region upstream of the LEC [40]. This possibility is supported by impairment in memory acquisition following damage to the perirhinal cortex in TEBC [5].

One may notice a trend that LEC-PrL disconnection had a larger effect compared with the unilateral LEC inactivation on remote retention, but not recent retention. This difference, however, was not statistically significant. Although our group size is comparable to our previous studies [6,14], a power analysis revealed our sample size has moderate power (54%) to detect an interaction between Group and Manipulation. This leaves open a possibility that some modifications of the design may allow us to detect the difference. 

The present result along with our previous study [6] suggests a long-lasting involvement of the LEC in the expression of memory in trace eyeblink conditioning. These findings resonate well with significant impairment in memory acquisition following large lesions of the entorhinal cortex (EC; [4]). In contrast, Suter, Weiss, and Disterhoft [5] reported that damage to the LEC does not impair acquisition in TEBC. Future studies are necessary to clarify what determines the necessity of LEC: the difference between acquisition and expression [5], the difference in the CS-US interval (500 ms vs. 250 ms), or the type of manipulation (reversible inactivation vs. permanent lesion). 

The present study demonstrates that the expression of recent and remote memory learned through TEBC depends on both hemispheres of the LEC, but not the PrL. These results provide further support for a long-lasting role of the LEC in memory expression. In addition, this study highlights the need for further studies with alternative methodologies to uncover the role of the LEC and its interaction with the PrL in TEBC. 
